# Off-Target Binding of Miglustat to Glycogen Debranching Enzyme

**DOI:** 10.3390/ijms27125490

**Published:** 2026-06-17

**Authors:** Drew Barber, Neha Mishra, Fiona Hegarty, Aviv Paz

**Affiliations:** 1 Hauptman-Woodward Research Institute, University at Buffalo, The State University of New York, Buffalo, NY 14203, USA; 2 Department of Structural Biology, Jacobs School of Medicine and Biomedical Sciences, University at Buffalo, The State University of New York, Buffalo, NY 14203, USA; 3 Smith College, Northampton, MA 01063, USA

**Keywords:** glycogen debranching enzyme, GDE, Miglustat, N-butyldeoxynojirimycin, iminosugars, glycogen metabolism, amylo-α-1,6-glucosidase

## Abstract

The iminosugar N-butyldeoxynojirimycin (Miglustat) is clinically used for the inhibition of ceramide glucosyltransferase for treating Type 1 Gaucher and Niemann–Pick type C diseases. This drug also inhibits glycogen debranching enzyme (GDE), the enzyme responsible for terminal glycogen catabolism via coordinated glucotransferase and amylo-α-1,6-glucosidase (GC) activities, although the structural basis for inhibition has been undefined. Here, we report the crystal structure of *Candida glabrata* GDE in complex with Miglustat, revealing inhibitor engagement at the conserved GC domain in an area that was previously hypothesized to accommodate the α-1,6-linked glucose moiety of glycogen. Structure-guided mutagenesis demonstrates that alanine substitution of residues at the GC site abolishes Miglustat binding, functionally validating the pocket and defining the interaction hot spots. To assess the possible relevance of these observations to the human enzyme, in silico docking predicts that Miglustat binds to the human enzyme in a pose close, albeit not identical, to our structure. These findings provide an opportunity to determine the molecular basis of GDE–inhibitor recognition, rationalize reported off-target effects of Miglustat, and provide a template for designing iminosugar therapies with reduced off-target binding.

## 1. Introduction

It is impossible to understate the importance of sugars to life on our planet. Simple sugar molecules or carbohydrates are used by almost all organisms as sources of energy and/or energy storage, through metabolic pathways like glycolysis, cellular respiration, and photosynthesis. Sugars are also fundamental components of nucleic acids, and are used to decorate and modify the activity of many proteins and lipids, modulating processes such as the immune response, cell signaling, and recognition. Thus, multiple proteins are responsible for the binding and processing of sugar molecules and some overlap is to be expected. Moreover, over 200 disorders of glycosylation caused by 189 genes have been identified [1].

One sugar-processing protein is glycogen debranching enzyme (GDE, EC 2.4.1.25 and EC 3.2.1.33), a four-domain enzyme that is conserved in animals and fungi [2]. Glycogen, the primary storage form of glucose in the body, is a branched starch molecule in which glucose residues are linearly linked through α-1,4 glycosidic bonds and branching of the chain occurs through α-1,6 glycosidic bonds [3,4]. In times of energetic needs, glucose molecules are cleaved off from the non-reducing ends of glycogen by the enzyme glycogen phosphorylase (GP). However, GP stops four residues prior to branching points [5]. To release these glucose moieties, the glucotransferase (GT, EC 2.4.1.25) domain of GDE breaks the branching points from the polymeric product of GP and transfers the resulting maltotriosyl group to a close by non-reducing end of glycogen, forming an α-1,4 glycosidic bond. Subsequently, the amylo-α-1,6, glucosidase (GC, EC 3.2.1.33) domain of GDE removes the remaining α-1,6-linked glucose residue from the branching point (Figure S1). Over 50 different mutations in GDE lead to Glycogen Storage Disease Type III (GSD III), a rare autosomal recessive disorder with an incidence of 1 in 100,000 that leads to accumulation of abnormal glycogen in organs such as the liver, and muscles, resulting in hepatomegaly, hypoglycemia, growth retardation, and, in many patients, progressive muscle weakness and cardiomyopathy [2,6,7]. These mutations were mapped on the structure of human GDE and the ones that cluster in the GT or GC domains significantly lower the activity of the enzyme and contribute to GSD III progression. Other mutations cluster in substrate-binding regions or areas that are thought to determine the structural integrity of GDE, as well as “other pathway” mutants that could not be explained by the existing structural and functional data [8]. A number of studies identified oligosaccharide binding sites in the Candida Glabrata GDE (CgGDE), spanning the GD, GC, and other accessory sites that are thought to be attachment sites for glycogen on the surface of the enzyme [2,9].

The iminosugar N-butyldeoxynojirimycin (Miglustat) is a glucose analog that is used therapeutically for the inhibition of ceramide glucosyltransferase (also called glucosylceramide synthase, GCS) in Type 1 Gaucher disease and Niemann–Pick type C disease, decreasing the accumulation and harmful buildup of glycosphingolipids in different organs. Due to the existence of multiple enzymes with glycosidase activities, Anderson et al. investigated the influence of a number of deoxynojirimycin derivatives on a number of such enzymes and found that Miglustat possesses an in vitro IC_50_ value of 4.5 µM towards GDE, but the binding site for Miglustat was not identified despite the existence of multiple sugar binding sites on GDE [10].

Several adverse effects reported during Miglustat therapy, including muscle weakness and fatigue, overlap clinically with the myopathic symptoms observed in Glycogen Storage Disease Type III (GSD III), caused due to GDE deficiency. Together with prior biochemical evidence demonstrating that deoxynojirimycin derivatives can inhibit the α-1,6-glucosidase activity of GDE, this clinical overlap suggests the possibility that off-target inhibition of GDE may contribute to certain neuromuscular effects associated with Miglustat treatment. Although no impact on glycogen breakdown by Miglustat treatment was predicted [10].

In this study, we outline the structural basis for Miglustat interaction with GDE by integrating X-ray crystallography, structure-guided mutagenesis, and ligand-binding assays in the fungal homolog. The crystal structure of CgGDE in complex with Miglustat reveals that the iminosugar binds within a conserved carbohydrate recognition site of the GC domain. Miglustat adopts a substrate mimetic pose stabilized by a characteristic network of polar hydrogen-bonding interactions that is distinct from ones previously reported for the binding of oligosaccharides at the GC site. Alanine substitutions at residues forming the binding pocket abrogate Miglustat binding, functionally validating the interaction surface.

To assess the relevance of these observations to the human enzyme, we performed in silico docking using DiffDock, which predicts that Miglustat preferentially occupies the conserved carbohydrate-binding pocket in both *Candida* and human GDE models. These findings are consistent with the high degree of conservation of active site residues and structural superposition between homologs, supporting a conserved mode of ligand recognition. These results identify the GC domain as the principal site of Miglustat recognition on GDE and provide a structural framework for understanding and potentially mitigating off-target interactions of sugar-based therapeutics with carbohydrate-processing enzymes.

## 2. Results

### 2.1. In Vitro Miglustat Binding to CgGDE

**Figure 1:**
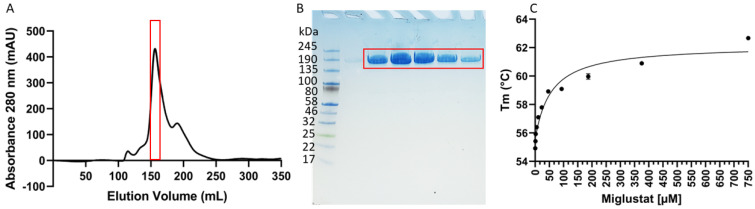
Purification of WT GDE and Miglustat binding analysis. (**A**) Size exclusion chromatogram of WT GDE using a High Load 26/60 Superdex 200 prep grade column. Red box denotes the fractions used for this study. (**B**) SDS-PAGE analysis of SEC peak fractions. (**C**) Miglustat-dependent thermal stabilization of WT GDE measured by nano differential scanning fluorimetry. The data fit was done using a one-site binding model by non-linear regression. Data were calculated as mean ± SD of three separate experiments each done in triplicate. The solid line represents the best fit to the one-site binding model.

It has been previously reported that iminosugars directly inhibit GDE, with 1-deoxynojirimycin (DNJ) selectively blocking the α-1,6-glucosidase activity of GDE and thereby leading to glycogenolysis arrest. Subsequent use of substrate-mimetic inhibitors indicated that six-membered DNJ-derived compounds, including N-alkylated derivatives such as Miglustat, retain the capacity to bind GDE through conserved carbohydrate recognition features [11,12,13,14,15]. Additionally, structural and biochemical studies have established that CgGDE recognizes α glucan-derived substrates through well-defined and highly conserved carbohydrate-recognition sites that can be inhibited by carbohydrate-mimetic glycosidase inhibitors [7]. However, the exact binding site and specific molecular interactions of Miglustat with GDE have not been established. Therefore, to evaluate the molecular basis of this interaction, full-length CgGDE was cloned with a C terminal hexahistidine tag, heterologously expressed in *Escherichia coli*, and purified to homogeneity, as confirmed by size exclusion chromatography and SDS–PAGE (Figure 1A,B).

At the time this study was initiated, no experimental human GDE structure was available. Consequently, CgGDE, which represents the closest structurally characterized homolog, was used as a validation system for mechanistic and structural investigation [2,7].

To validate and characterize the binding of Miglustat to CgGDE, nano differential scanning fluorimetry (nanoDSF) was performed to reveal a concentration-dependent increase in the thermal stability of CgGDE upon Miglustat binding (Figure 1C) suggesting a specific binding interaction in which Miglustat stabilizes CgGDE, a hallmark of small molecule engagement observed by thermal shift assays. Fitting the dose-dependent titration to a single-site binding model yielded an apparent Kd value of 56 µM, consistent with a specific, saturable interaction between Miglustat and CgGDE.

This apparent Kd falls within the micromolar range reported for inhibition of recombinant human glycogen debranching enzyme, with an IC_50_ of 4.5 µM measured in vitro [10], and is also comparable to the concentration range (IC_50_ of 5–50 µM) reported for biochemical inhibition of human GCS, the primary therapeutic target of Miglustat, used for the treatment of Gaucher disease type I [16].

### 2.2. CgGDE–Miglustat Complex Structure

**Figure 2:**
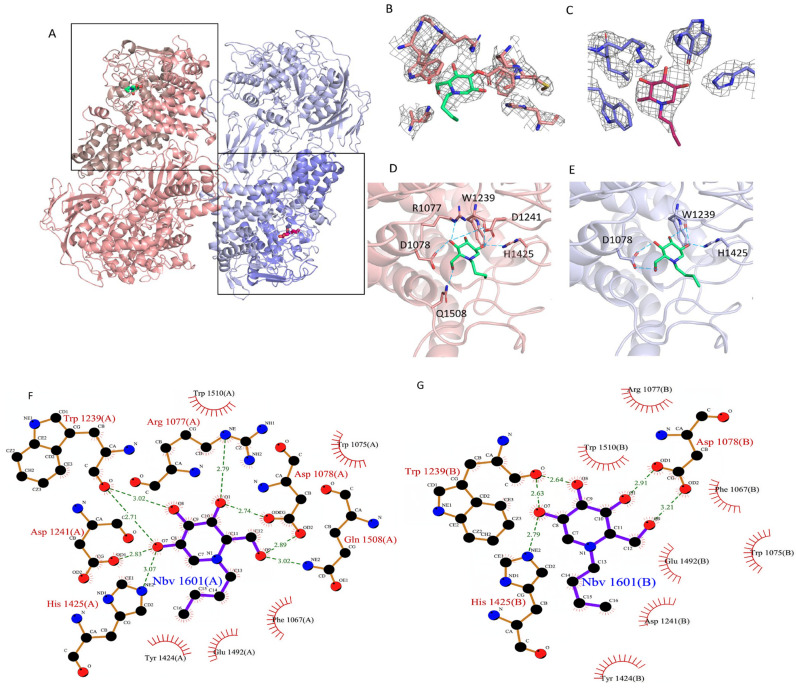
Structural analysis of the GDE GC catalytic pocket and Miglustat binding. (**A**) A cartoon representation of the GDE–Miglustat complex highlighting the GC-catalytic site. (**B**,**C**) Electron density maps for CgGDE–Miglustat crystal structure (11TN). (**B**) 2Fo-Fc maps for the ligand-bound structure shown as cartoon for chain A (shown in salmon color) and (**C**) chain B (blue color) at σ = 1.5 (blue mesh) illustrating clear and continuous electron density for the ligand. Miglustat is shown as green and pink sticks bound to chain A and chain B respectively. (**D**,**E**) Miglustat binding within the GC catalytic pocket in chain A (**D**) and chain B (**E**). Miglustat is shown as sticks, and interacting residue side chains are displayed as sticks and labeled, highlighting key polar and electrostatic residues involved in ligand binding. Hydrogen bonds are shown as broken cyan lines. Miglustat adopts a similar binding mode in both chains. (**F**,**G**) LigPlot interaction diagrams for chain A (**F**) and chain B (**G**), highlighting hydrogen-bonding interactions and hydrophobic contacts.

To define the molecular basis underlying the association of Miglustat with GDE, we determined the crystal structure of CgGDE in complex with Miglustat (Figure 2A). Crystals of the complex were obtained by co-crystallization, and X-ray diffraction data were collected and processed to a resolution of 3.24 Å (Table 1). The structure was refined to an acceptable stereochemical quality (Table 2). Regions of chain B lacking interpretable electron density were omitted during model building and refinement.

Clear and continuous electron density was observed for Miglustat within the GC domain of each protomer, enabling placement of the ligand within the catalytic pocket. Due to the medium resolution of the structure, multiple Miglustat-binding poses were docked and refined independently in both chains, and the pose showing the best agreement with the electron density, real space correlation, and stereochemical quality was selected for each protomer. Importantly, the best binding poses in the two protomers are almost identical. The ligand placement is supported by complementary electron density analyses. 2Fo–Fc maps (Figure 2B,C) define the overall shape and continuity of the ligand within the binding site, while Fo–Fo difference maps reveal ligand-specific density relative to the apo structure (PDB: 5D06) under identical crystallization conditions (Figure S2A,B). POLDER maps further confirmed the presence and orientation of Miglustat in the GC catalytic site of GDE (Figure 2D and Figure S2C). Despite this supporting evidence, it remains possible that the exact conformations of Miglustat within the binding pockets of the two chains differ slightly from the modeled structures, owing to the medium resolution of the diffraction data.

The CgGDE–Miglustat complex shows that the six-membered iminosugar occupies the highly conserved GC catalytic domain of GDE which is involved in substrate recognition. This region of CgGDE is spanned by residues 1067–1508. Consistent with the role of GDE in recognizing carbohydrate-derived substrates, the binding pocket was observed to be predominantly polar in nature. The iminosugar makes a network of hydrogen-bonding and non-bonded interactions including seven hydrogen bonds in chain A and five in chain B, together with extensive non-bonded contacts, indicating tight coordination of Miglustat in both protomers (Figure 2D,E). Asp1078 acts as the primary electrostatic anchor in chains A and B. Both of its side-chain oxygens form hydrogen bonds with the O1 and O9 oxygens of Miglustat. Other common residues forming hydrogen bonds in both protomers are Trp1239 in which its main chain oxygen forms hydrogen bonds with Miglustat’s O7 and O8, and His1425 in which N^ε2^ forms a hydrogen bond with O7 in Miglustat (Figure 2F,G). Due to a slight shift in the position of Miglustat between the protomers, in chain A there are additional hydrogen bonds, namely Arg1077 N^ε^ with Miglustat’s O1, Asp 1241 O^δ1^ 1 with O7 in Miglustat, and Gln1508 N^ε2^ with O9 in Miglustat. Aromatic residues lining the active site, Phe1067, Trp1075, Trp1239, Tyr1424, and Trp1510 participate in non-bonded contacts that further confine the ligand within the catalytic site, restricting ligand mobility and thereby enhancing shape complementarity.

**Figure 3:**
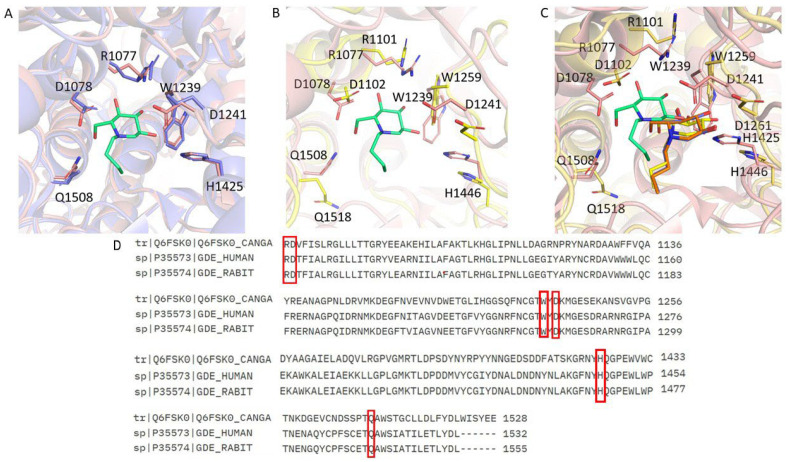
Structural and evolutionary conservation of the GC catalytic domain across *Cg* and human GDE. (**A**) Close-up view of the catalytic pocket in the crystal structure of apo *Cg*GDE (PDB ID: 5D06) colored in blue and the Miglustat-bound structure colored in salmon, highlighting conserved residues contributing to the active-site environment. Miglustat is shown as green sticks. Key residue side chains are displayed as sticks and labeled. (**B**) GC catalytic sites of human GDE (PDB ID: 8ZEQ) colored in yellow, and the Miglustat-bound *Cg*GDE colored in salmon demonstrating conservation of the catalytic pocket architecture. The experimentally determined Miglustat is shown in green sticks. (**C**) Superposition of DiffDock human GDE–Miglustat docking colored in yellow with *Cg*GDE–Miglustat crystal structure. The *Cg*GDE-bound Miglustat is shown as green sticks while the predicted docking poses are shown in yellow, orange, and blue. (**D**) Multiple sequence alignment of GDE from *Candida glabrata*, human, and rabbit, highlighting conserved residues within the GDE GC catalytic pocket. Conserved residues are outlined in red boxes, emphasizing strong evolutionary conservation of this functionally important site.

Comparison of our structure with that of apo CgGDE (PDB: 5D06) showed that Miglustat binding does not induce any significant conformational rearrangements (Figure 3A). Cg GDE bound to maltopentaose (PDB ID: 5D0F) demonstrates that Miglustat binds close to the site identified for maltopentaose (Figure S3A) but closer to the main protein mass, occupying a volume that is compatible with the putative location of the α 1,6 branched sugar (Figure 3A). Figure S3B,C show the same trend for the location of the sugars for two other structures with bound sugars in the GC domain: PDBID 7EIM for maltopentaose and PDBID 7EKX for maltononaose.

Structural superposition with the ligand-free human GDE (PDB ID: 8ZEQ) further demonstrates strong conservation of the GC domain architecture (Figure 3B) with the Cg protein. In addition, sequence alignment across fungal and mammalian GDEs reveals that residues defining the Miglustat-binding pocket/GC catalytic site are highly conserved (Figure 3D). Consistent with the sequence and structural similarities, DiffDock-based docking [17] of Miglustat into the structure of ligand-free human GDE predicted that 3 out of the 10 high-rank docking poses for Miglustat are at the conserved GC site close to the pose we identified for CgGDE (Figure 3C and Figure S4). Table S1 lists the PDB IDs of deposited GDE structures used for this comparison.

Together, these observations support a model in which the iminosugar associates with the pre-existing α 1,6 branched carbohydrate-binding site of the GC domain, consistent with previously observed oligosaccharide-binding modes [16].

### 2.3. Binding Site and Thermal Shift Analyses

Our structural analysis revealed Miglustat binding to CgGDE in the GC catalytic cleft and the specific residues interacting with the drug. We therefore evaluated the functional basis of this interaction using structure-guided mutagenesis. Since there are multiple interactions between Miglustat and side-chains at the site, a GC-site mutant in which multiple residues forming the binding interface (Arg1077, Asp1078, Asp1241, Glu1492, and Gln1508) substituted to alanine was generated.

The effect of the GC-site mutant on the stability of the protein was assessed using thermal shift analysis by differential scanning fluorimetry (DSF) with SYPRO Orange dye (Thermo Fisher Scientific, Waltham, MA, USA) (Figure S5A). Wild-type GDE exhibited an increase in apparent melting temperature (Tma) from 53.5 °C in the apo state to 58.3 °C at a saturating Miglustat concentration (Figure S5B), whereas the mutant displayed a lower Tma for the apo condition at 50 °C suggesting some destabilization upon the GC-site mutation. Importantly, the Tma did not shift to a higher temperature upon incubation with saturating concentrations of Miglustat (Figure S5C,D) suggesting a complete loss of Miglustat binding to the mutant, demonstrating that the disruption of the GC catalytic cleft is sufficient to abolish the iminosugar association with CgGDE.

Altogether, the structural and functional dose-dependent Miglustat binding analyses confirm that the iminosugar binding is mediated by a specific, catalytically relevant interaction surface within the GC domain rather than non-specific binding. Miglustat stabilizes the wild-type GDE by binding to a conserved polar interaction network, and disruption of the key pocket interacting residues impairs the ligand-induced stabilization. This is also in agreement with past findings that showed inhibition of GDE activity by Miglustat; although the previous reports established functional inhibition in vitro [10], these studies did not resolve domain specificity within GDE or the structural determinants for the binding mechanism. While several other iminosugars are known to inhibit glycosidases [18], our data directly links this inhibition with the GC-specific binding of GDE with Miglustat.

## 3. Discussion

The GDE–Miglustat complex structure reveals that Miglustat binds directly within the canonical GC catalytic cleft of the enzyme, exploiting a highly conserved, polar carbohydrate-recognition environment. The iminosugar ring is anchored within this site by key hydrogen bonds and electrostatic interactions involving residues associated with glucan substrates and the binding occurs without substantial conformational rearrangement of the enzyme, indicating Miglustat occupies a pre-organized active site rather than inducing a new conformation to the inhibitory pocket.

Substitution of the key interacting residues to alanine abolished Miglustat binding, highlighting the functional relevance of these contacts. This confirms that inhibition depends on a well-defined catalytic interaction network rather than non-specific binding. This finding indicates that Miglustat acts as a competitive-like, non-productive inhibitor by occupying the catalytic core of the enzyme while lacking the extended glucan features required for catalysis.

Miglustat is well recognized as a relatively broad-spectrum iminosugar, with its known interactions with multiple carbohydrate-processing enzymes in addition to its bona fide target, GCS. Previous studies have shown that, in addition to GCS, Miglustat can interact with a range of glycoenzymes, including ER α glucosidases I and II, the non-lysosomal β glucosidase GBA2, and intestinal disaccharidases [19,20,21,22,23]. Consistent with this, Miglustat exhibits inhibitory activity across these enzymes over a broad potency range, from submicromolar inhibition of intestinal disaccharidases (e.g., sucrase and maltase) to micromolar inhibition of glucosylceramide synthase (~5–50 µM) and β-glucosidase 2 (GBA2) (12–310 nM). The apparent Kd for CgGDE (~56 µM) falls within this reported micromolar range. Table S2 lists the relative potency of Miglustat towards the different targets, suggesting that GDE represents a plausible, though not dominant, off-target interaction. Direct comparisons across studies should, however, be interpreted with caution due to differences in assay formats. The off-target interactions of Miglustat arise from the iminosugar scaffold’s ability to mimic carbohydrate substrates and transition states, rather than from target-specific optimization. This behavior contrasts with ceramide-mimetic inhibitors such as eliglustat, which achieve higher target specificity by engaging lipid-binding determinants of GCS rather than broadly conserved carbohydrate-recognition sites. The superposition of the GC site in our Miglustat-bound structure with GDE lacking bound sugars at the GC site (PDB: 5D06) and the apo human enzyme further supports this view, as only minimal changes are observed in the overall geometry of the catalytic cavity and the positioning of key active site residues.

While iminosugar binding to carbohydrate-active enzymes is well documented, and structures of non-branched sugars bound at the GC site have been reported, the present structure offers a molecular snapshot of interactions within this region. In particular, it highlights how the conserved α-1,6 glucan-processing motif in the GDE family can accommodate sugar-like ligands and reveals a network of surrounding side chains that may be positioned to interact with α-1,6 glucans. Taken together, these observations support a broader principle that has emerged from prior work on iminosugars: carbohydrate-binding motifs across glycoenzyme families can contribute to limited selectivity and off-target engagement. Rather than suggesting the emergence of a dominant off-target pathway, these findings extend the principle to the need to consider distinct sugar binding sites when interpreting pharmacological effects and in the development of next-generation iminosugar-based therapeutics.

In a wider context, our findings are consistent with growing efforts in the drug discovery community to systematically address off-target and anti-target interactions through dedicated frameworks such as the recently proposed “avoidome” [24]. This concept emphasizes the proactive identification of conserved binding environments that represent predictable liabilities for small molecule scaffolds, particularly those designed to mimic endogenous metabolites. The ability of Miglustat to engage GDE through a conserved catalytic architecture illustrates how off-target binding can arise from well-defined and structurally rational interactions rather than non-specific promiscuity. By providing a detailed example of such engagement, the present structure supports the view that integrating structural information on off-target susceptibility early in lead optimization may be essential for improving drug selectivity and minimizing late-stage safety liabilities. Our findings provide a framework for understanding iminosugar interactions; however, further studies integrating orthogonal biophysical approaches and functional assays will be important to refine the quantitative and physiological relevance of this interaction, particularly in the context of the human enzyme.

## 4. Materials and Methods

### 4.1. Materials

CgGDE was cloned into the pET 15b expression vector and transformed into *Escherichia coli* BL21 (DE3) competent cells for recombinant production. Luria–Bertani broth served as the culture medium and was supplemented with ampicillin at a final concentration of 100 µg mL^−1^. Isopropyl β D 1 thiogalactopyranoside (IPTG) was used for induction at a final concentration of 0.3 mM. Buffer components included Tris–HCl, NaCl, β mercaptoethanol (βME), dithiothreitol (DTT), tris (2 carboxyethyl) phosphine (TCEP), glycerol, and imidazole at the concentrations specified below. Miglustat (2R,3R,4R,5S)-1-Butyl-2-(hydroxy methyl) piperdine-3,4,5-triol) (Ambeed, Arlington Heights, IL, USA) was prepared as a 20 mM stock solution in water and stored at −20 °C prior to serial dilution for binding and crystallization experiments. Affinity purification employed Ni–NTA resin (HisPure, Thermo Fisher Scientific, Waltham, MA, USA), followed by reverse Ni–NTA using a HisTrap column (Cytiva, Marlborough, MA, USA) on an ÄKTA Pure chromatography system (Cytiva, Marlborough, MA, USA). Size exclusion chromatography (SEC) was performed on a HiLoad 26/60 Superdex 200 pg column (Cytiva, Marlborough, MA, USA). NanoDSF measurements were acquired on a Prometheus Panta instrument (NanoTemper Technologies, Munich, Germany).

### 4.2. Cloning of Glycogen Debranching Enzyme

The CgGDE gene, codon-optimized for expression in *E. coli*, was synthesized by VectorBuilder (Chicago, IL, USA) and restriction-cloned into a pET15b vector using NdeI and BamHI restriction sites. All mutants were created using site-directed mutagenesis and sequences were verified by Eurofins Genomics. Cloning and propagation were carried out in XL10-Gold cells (Agilent Technologies, Santa Clara, CA, USA).

### 4.3. Protein Expression and Purification

For the wild-type enzyme, *E. coli* BL21 (DE3) transformed with pET 15 GDE was cultured in LB medium containing 100 µg mL^−1^ ampicillin at 37 °C with shaking until the optical density at 600 nm reached approximately 0.6. Protein expression was induced by adding IPTG to 0.3 mM, after which cultures were shifted to 16 °C and incubated for 12–14 h. Cells were harvested at 3500 rpm for 10 min at 4 °C using a JLA 8.1 Beckmann centrifuge (Beckman Coulter, Brea, CA, USA), and the pellets were resuspended in lysis buffer consisting of 20 mM Tris–HCl (pH 7.5), 300 mM NaCl, and 2 mM βME. Cells were lysed by three passes through a pre-equilibrated microfluidizer operated at 16,000 psi. The lysate was clarified by centrifugation at 15,000 rpm for 30 min at 4 °C, and the resulting supernatant was subjected to immobilized metal affinity chromatography. The soluble fraction was incubated with Ni–NTA beads at 4 °C for 1 h with gentle mixing, washed with lysis buffer containing 20 mM imidazole, and eluted using the same buffer supplemented with 400 mM imidazole. Eluted protein was pooled and dialyzed overnight at 4 °C in the presence of TEV protease at a 1:20 (*w*/*w*) TEV:protein ratio against 20 mM Tris–HCl (pH 7.5), 200 mM NaCl, 2 mM DTT, and 5% (*v*/*v*) glycerol to remove the N terminal affinity tag. The dialyzed protein was then applied to a HisTrap column on an ÄKTA Pure system for reverse Ni–NTA chromatography purification to remove uncleaved protein, His-tagged contaminants, and TEV protease; the cleaved, tag-free GDE was collected in the flow through. Final polishing was performed by SEC on a HiLoad 26/60 Superdex 200 pg column equilibrated in 20 mM Tris–HCl (pH 7.5), 200 mM NaCl, 2 mM DTT, and 5% glycerol, with fractions monitored by UV absorbance and SDS–PAGE. Peak fractions were pooled, concentrated, and flash-frozen and stored at −80 °C. Protein purity was assessed by SDS–PAGE, concentration was determined from A_280_ using the calculated extinction coefficient, and monodispersity was evaluated from the size-exclusion chromatography (SEC) elution profile.

The mutant GDE was expressed and purified following the same protocol with minor changes. Cultures of 1 L LB were induced with 0.3 mM IPTG, harvested at 4000× *g* for 10 min at 4 °C, and resuspended in 20 mM Tris–HCl (pH 7.5), 300 mM NaCl, 5% (*v*/*v*) glycerol, and 2 mM DTT prior to lysis. Downstream purification steps were identical to those of the wild-type protein, including IMAC, TEV cleavage with dialysis, reverse Ni–NTA, and SEC under equivalent buffer conditions.

### 4.4. Crystallization and Data Collection

For co-crystallization with Miglustat, purified wild-type GDE was concentrated to 20 mg mL^−1^ and incubated with 500 µM ligand. Immediately before setting crystallization drops, the protein–ligand mixture was supplemented with 0.3 mM TCEP. Crystals were obtained using vapor diffusion under conditions comprising 0.1 M MES (pH 7.0), 7% (*v*/*v*) Tacsimate (pH 7.0), and 20% (*w*/*v*) PEG MME 5K. Drops were set up as sitting drop at room temperature. Crystals were cryoprotected and flash cooled in liquid nitrogen prior to data collection.

X-ray diffraction data were collected at the National Synchrotron Light Source II (NSLS II) AMX beamline (17 ID 1) at Brookhaven National Laboratory. Diffraction data were indexed and integrated, then scaled, merged, and analyzed for anisotropy using STARANISO [25] (Global Phasing Ltd., Cambridge, UK). Ellipsoidal truncation was applied based on anisotropic resolution limits, with parameters summarized in Table 1. The resulting refined structure has been deposited in the Protein Data Bank under accession code 11TN.

### 4.5. Structure Determination and Refinement

Phasing was achieved by molecular replacement using Phaser [26], with an AlphaFold [27] predicted model of CgGDE serving as the search model since the statistics of this search were superior to searches using deposited GDE structures. Iterative model building was conducted in Coot [0.9.8.96], and restrained refinement was performed in REFMAC5 [5.8.0431] [28]. Ligand coordinates and restraint dictionaries for Miglustat were obtained from the NBV ligand library and used for refinement. TLS and non-crystallographic symmetry restraints were applied during model refinement [29]. Model validation employed MolProbity [30], and ligand placement was justified against 2mFo–DFc and mFo–DFc maps, with composite polder maps prepared as an additional check for model bias [31]. Final refinement statistics, including resolution range, the number of reflections for work and free sets, Rwork and Rfree values, numbers of atoms per component class, average B factors, root mean square deviations for bonds and angles, Ramachandran metrics, and clashscore are presented in Table 2.

### 4.6. Binding Studies and Binding Site Analysis

Ligand binding and thermal stability were assessed by nanoDSF using a Prometheus Panta instrument. For wild-type GDE, peak size exclusion chromatography fractions were adjusted to a final protein concentration of 500 nM and incubated with Miglustat at final concentrations of 0, 1, 2, 5, 11, 23, 46, 93, 187, 375, and 750 µM. All protein–ligand mixtures were incubated for 30 min at 4 °C prior to measurement. Thermal unfolding profiles were recorded over a defined temperature gradient, and Tma were determined from the first derivative of the fluorescence ratio (F350/F330). Each condition was measured in triplicate and the experiment was independently repeated three times. For the Miglustat titration experiments, ligand-dependent thermal stabilization was quantified by fitting the concentration-dependent Tm shifts using non-linear regression assuming a one-site binding model.

Differential scanning fluorimetry (DSF) was performed using a Bio-Rad CFX real-time PCR instrument in the size exclusion buffer. Measurements were carried out in 96-well plates with a total volume of 25 µL per well. Wild-type and mutant GDE samples were used at a final concentration of 1.4 µM, with or without 1 mM Miglustat, following a 30 min incubation at 4 °C prior to analysis. SYPRO Orange was diluted from a 5000× stock to the working concentration of 5× in the corresponding buffer and added immediately prior to the experiment to minimize photobleaching.

Thermal denaturation was monitored by increasing the temperature from 25 °C to 95 °C at a controlled ramp rate, while fluorescence was recorded as a function of temperature. The resulting unfolding profiles were analyzed using DSFworld software (accessed on 22 April 2026) [32], and Tma were determined from the peak of the first derivative (dRFU/dT) of the fluorescence curves. Each condition was measured in triplicate.

Structural analysis of the Miglustat-binding interface was performed using the refined crystal structure. Protein–ligand interactions, including hydrogen bonds, hydrophobic contacts, and other noncovalent interactions, were identified and analyzed using PDBsum [33]. Structural visualization and inspection were carried out using PyMOL 3.1.5.1 (Schrödinger, LLC, New York, NY, USA).

Molecular docking of Miglustat was performed to assess the conservation of the binding mode observed in the crystal structure and to evaluate its relevance to the human enzyme. Docking calculations were carried out using DiffDock 1.1.3 [15], a deep learning-based docking framework that predicts ligand-binding poses without requiring predefined binding sites.

Docking was performed using default parameters on the global protein to generate multiple candidate binding poses, which were ranked by confidence score. The top poses were analyzed for consistency with the experimentally observed binding site and inspected in PyMOL. For human GDE, poses located within the conserved GC catalytic pocket were considered indicative of a conserved binding mode.

## Figures and Tables

**Table 1:** Data collection and processing.

Wavelength	0.92010 Å
Space group	C2221
a, b, c	159.078, 199.274, 254.213
α, β, ϒ	90.0, 90.0, 90.0
Resolution range	124.323–3.236 (3.595–3.236)
No. of unique reflections	36,604 (1830)
Completeness (%) spherical/ellipsoidal	56.7/93.5
Mulitplicity	14.2 (13.5)
(I/σ)	6.2 (1.5)
CC1/2	0.985 (0.527)

Values in parentheses refer to the highest resolution shell.

**Table 2:** Refinement statistics.

Resolution (Å)	127.11–3.24
(I/σ)	1.85 (at 3.26 Å)
Refinement program	REFMAC 5.8.0431
R_work_/R_free_	0.235/0.274
R free test set	1857 reflections (2.88%)
Wilson B-factor (Å^2^)	64.5
Anisotropy	0.166
Estimated twinning fraction	No twinning to report
Fo, Fc correlation	0.87
Average B, all atoms	87
PDB code	11TN

## Data Availability

The data presented in this study are openly available in the PDB at https://www.rcsb.org/, accession number 11TN.

## Supplementary Materials

The following supporting information can be downloaded at: https://www.mdpi.com/article/10.3390/ijms27125490/s1.

## Abbreviations

The following abbreviations are used in this manuscript:

CgGDE	Candida glabrata glycogen debranching enzyme
GC	Amylo-α-1,6-glucosidase domain
GCS	Glucosylceramide synthase
GDE	Glycogen debranching enzyme
GP	Glycogen phosphorylase
GT	Glucanotransferase domain
GSD III	Glycogen storage disease type III
Miglustat	N-butyldeoxynojirimycin
IC_50_	Half maximal inhibitory concentration
MR	Molecular replacement
PEG MME 5K	Polyethylene glycol monomethyl ether (5000 Da)
SEC	Size exclusion chromatography
TEV	Tobacco etch virus protease
TCEP	Tris-(2-carboxyethyl)-phosphine
βME	β-Mercaptoethanol
nanoDSF	Nano differential scanning fluorimetry
DSF	Differential scanning fluorimetry
