# Supplementary material for: Off-Target Binding of Miglustat to Glycogen Debranching Enzyme
Source: Int J Mol Sci. 2026 Jun 17;27(12):5490. doi: 10.3390/ijms27125490 (PMC13299489; doi:10.3390/ijms27125490)
Supplement: Supplementary file 1 [file ijms-27-05490-s001.zip › ijms-4308514-supplementary.pptx]

## Slide 1
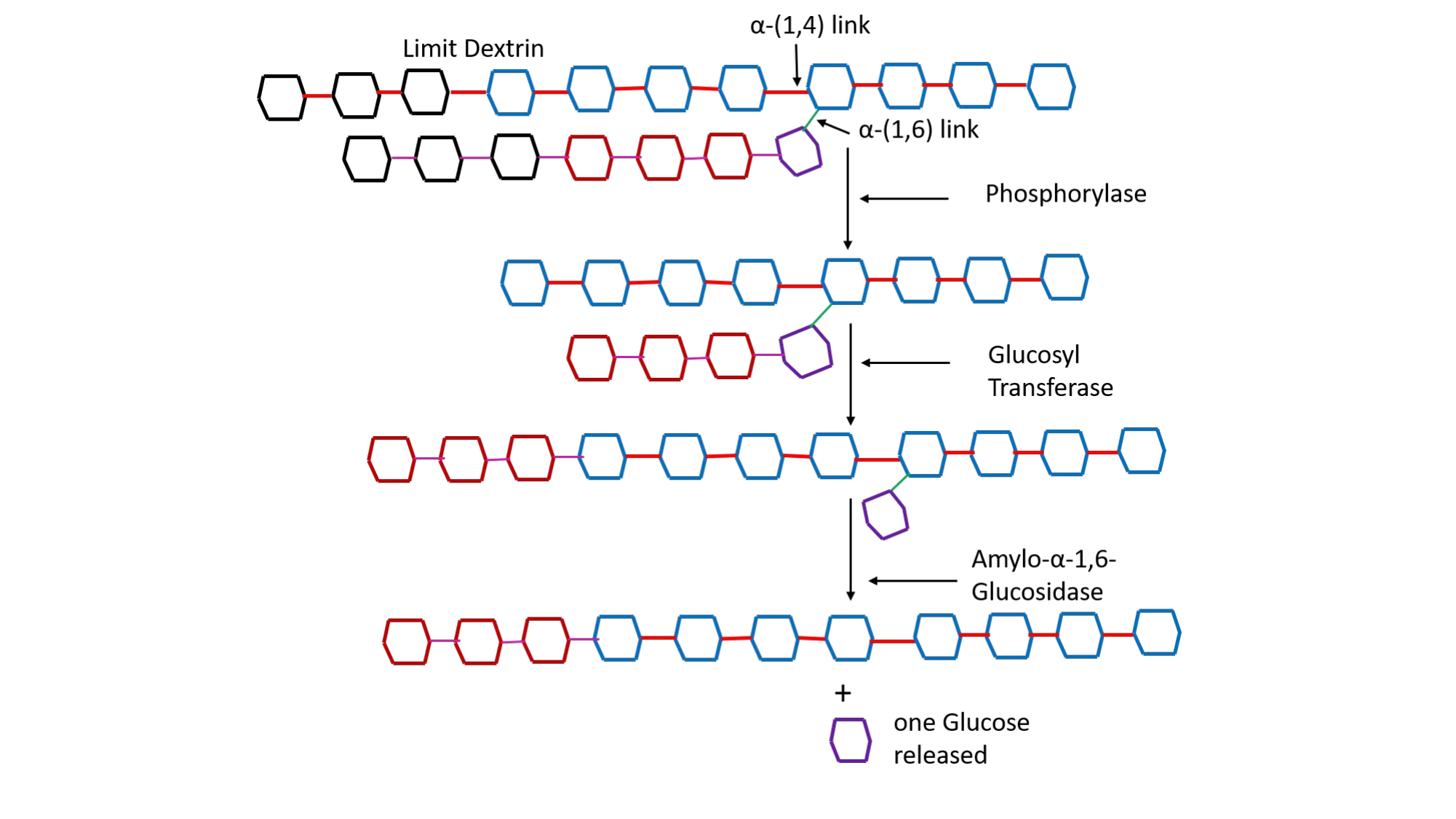

## Slide 2
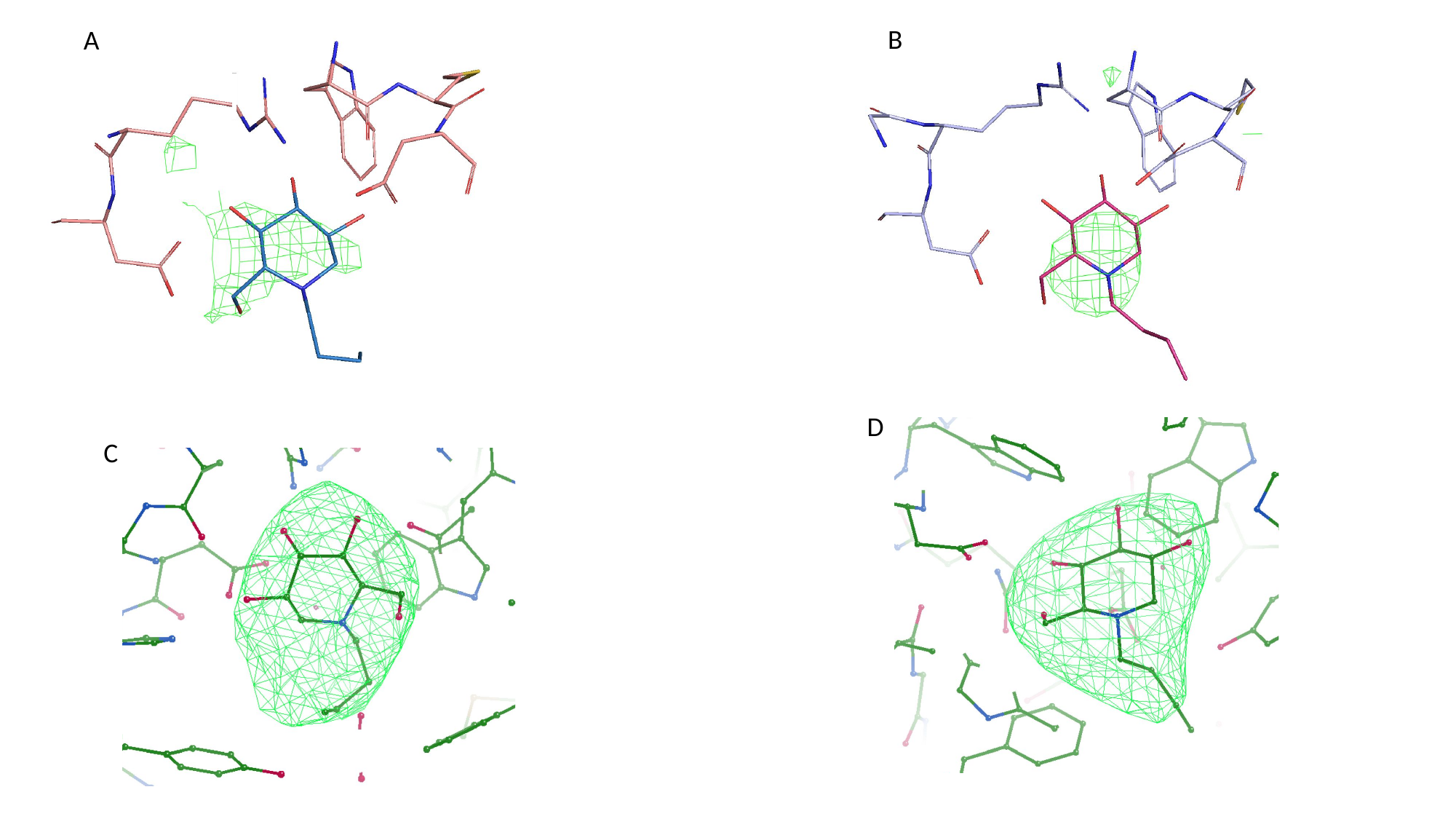

B
A
D
C

## Slide 3
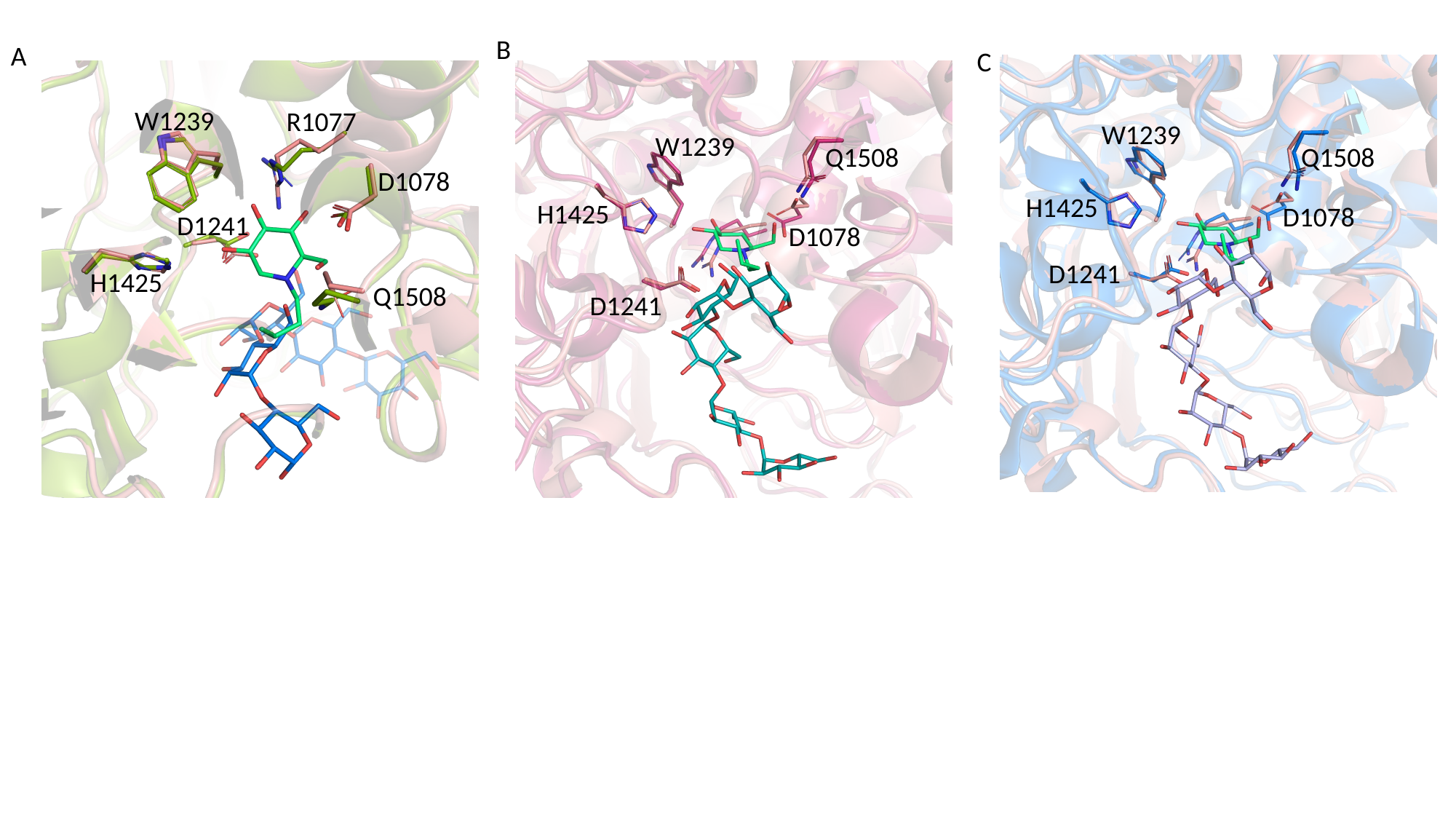

B
A
C
W1239
Q1508
H1425
D1078
D1241
W1239
Q1508
H1425
D1078
D1241
W1239
R1077
D1078
D1241
H1425
Q1508

## Slide 4
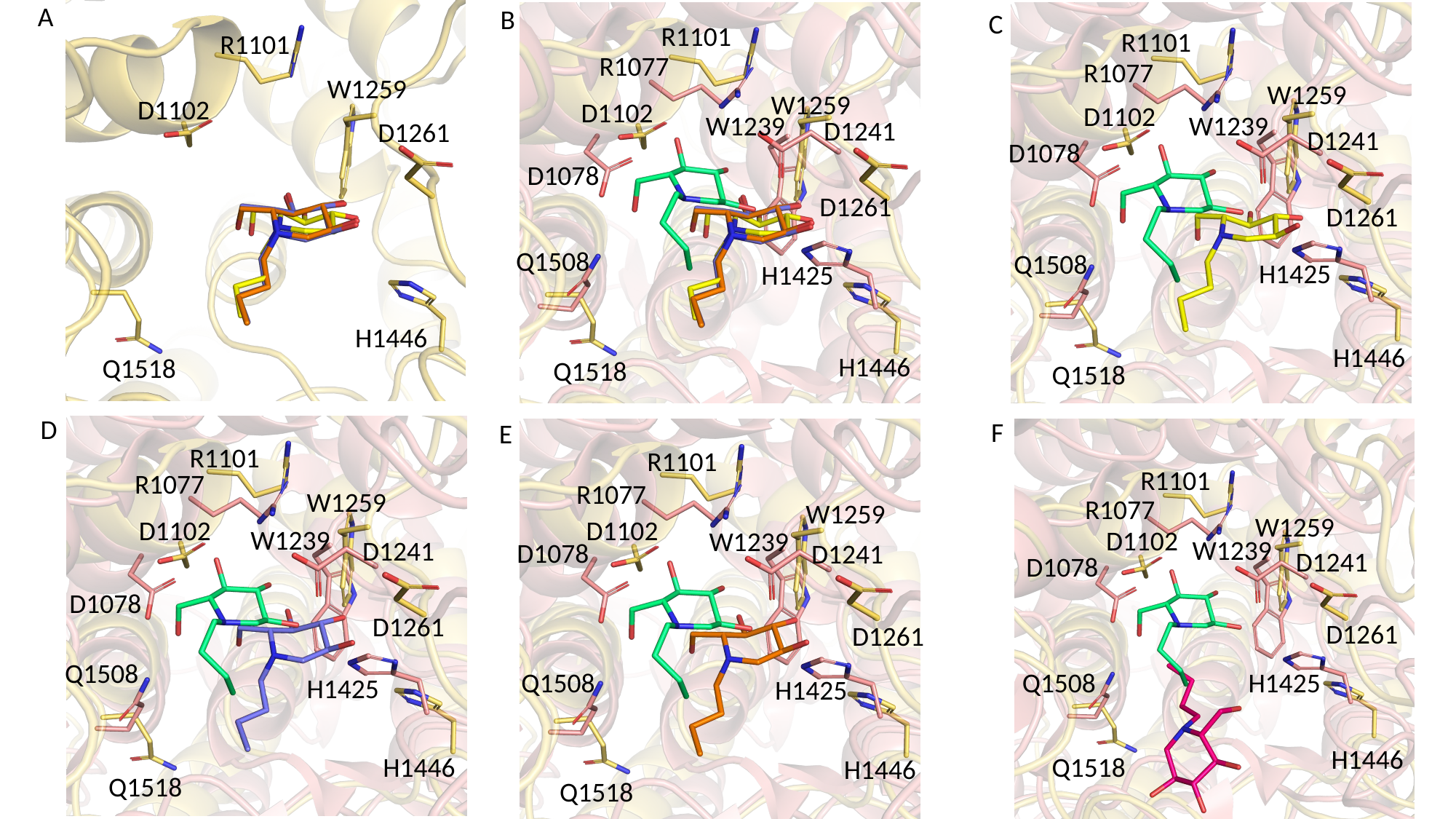

A
B
R1101
W1259
D1102
D1261
H1446
Q1518
C
R1101
R1077
W1259
D1102
W1239
D1241
D1078
D1261
Q1508
H1425
H1446
Q1518
R1101
R1077
W1259
D1102
W1239
D1241
D1078
D1261
Q1508
H1425
H1446
Q1518
D
F
E
R1101
R1077
W1259
D1102
W1239
D1241
D1078
D1261
Q1508
H1425
H1446
Q1518
R1101
R1077
W1259
D1102
W1239
D1078
D1241
D1261
Q1508
H1425
H1446
Q1518
R1101
R1077
W1259
D1102
W1239
D1241
D1078
D1261
Q1508
H1425
H1446
Q1518

## Slide 5
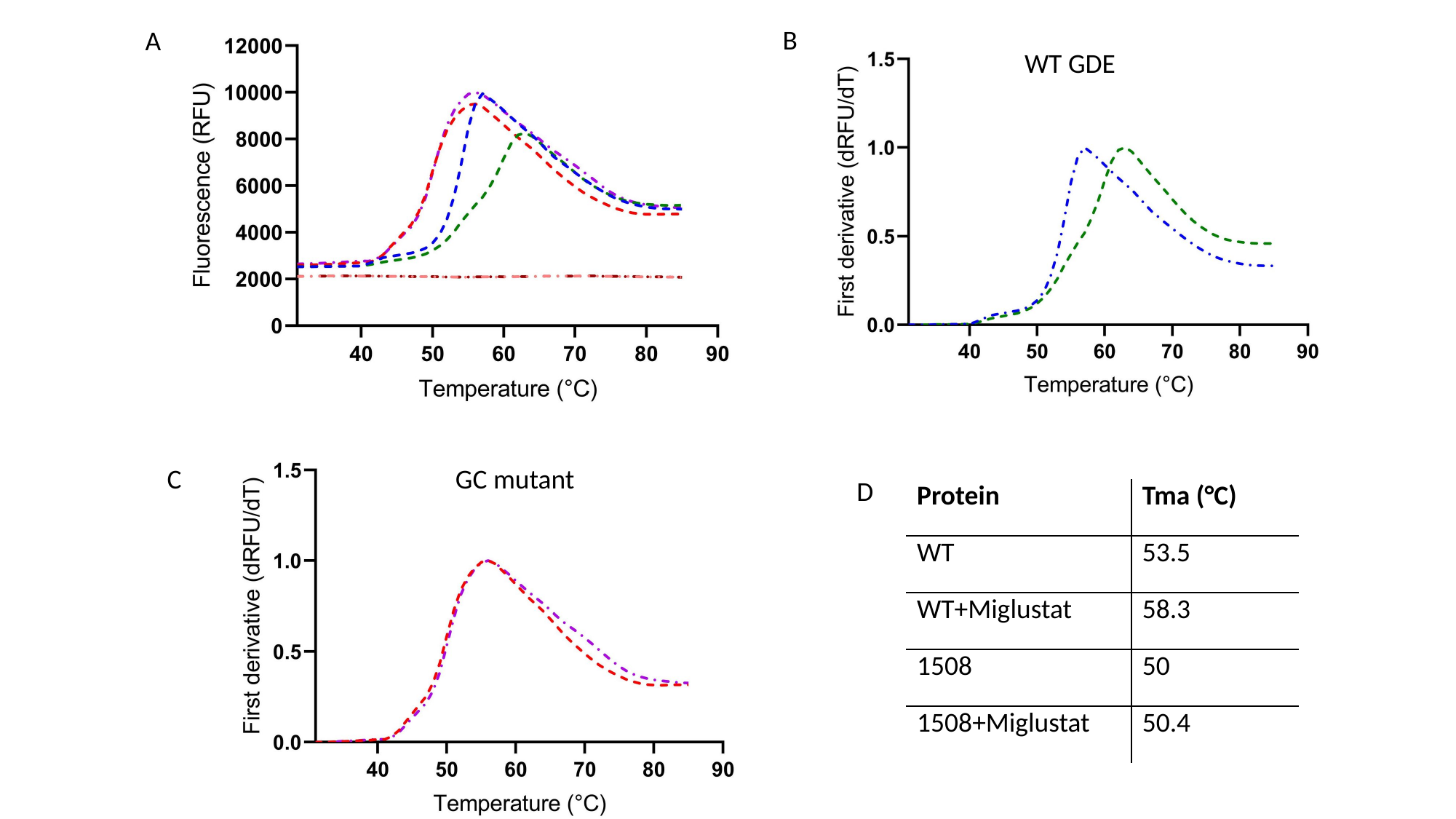

B
A
WT GDE
C
GC mutant
D
| Protein | Tma (°C) |
| --- | --- |
| WT | 53.5 |
| WT+Miglustat | 58.3 |
| 1508 | 50 |
| 1508+Miglustat | 50.4 |

## Slide 6
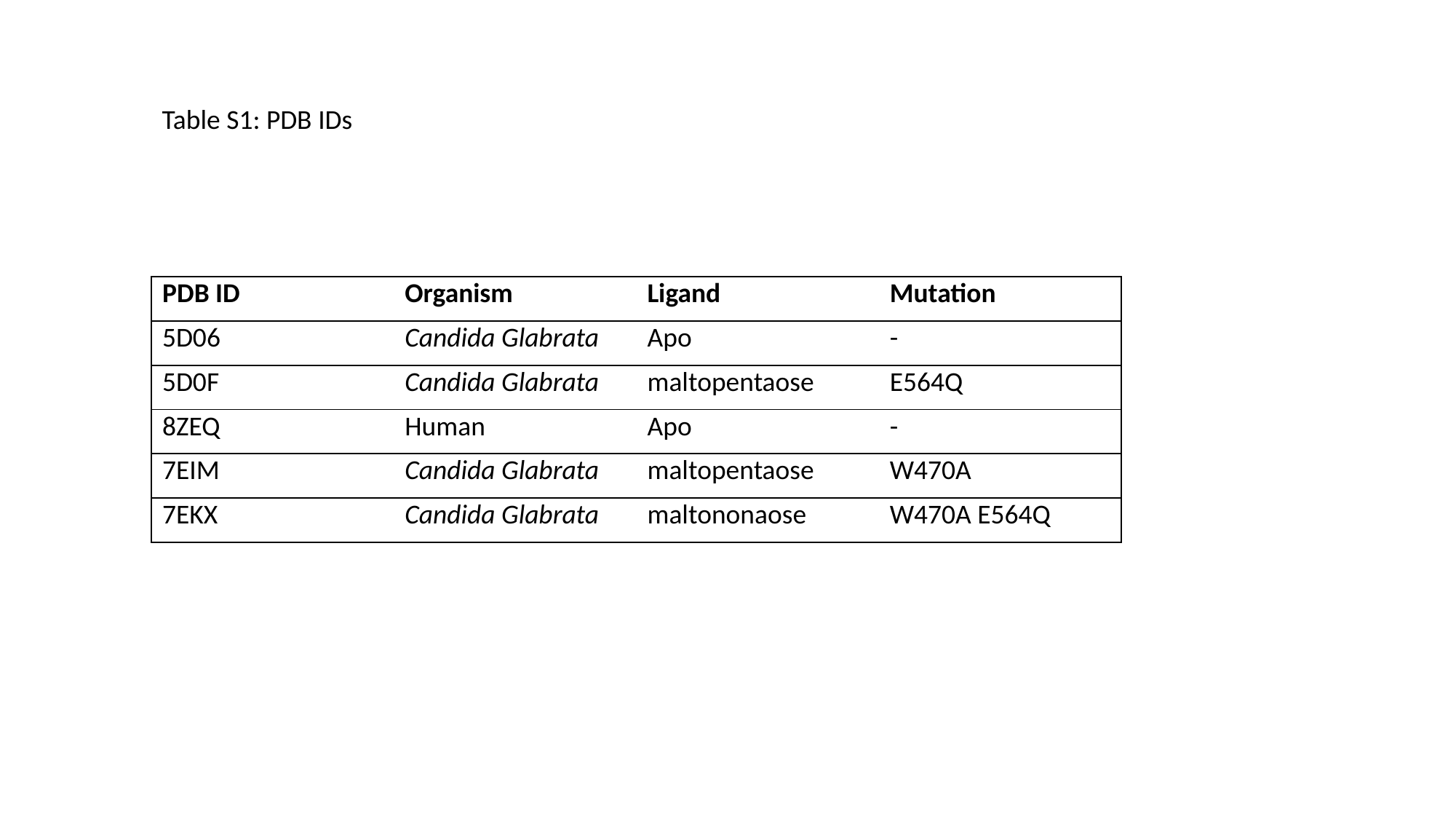

Table S1: PDB IDs
| PDB ID | Organism | Ligand | Mutation |
| --- | --- | --- | --- |
| 5D06 | Candida Glabrata | Apo | - |
| 5D0F | Candida Glabrata | maltopentaose | E564Q |
| 8ZEQ | Human | Apo | - |
| 7EIM | Candida Glabrata | maltopentaose | W470A |
| 7EKX | Candida Glabrata | maltononaose | W470A E564Q |

## Slide 7
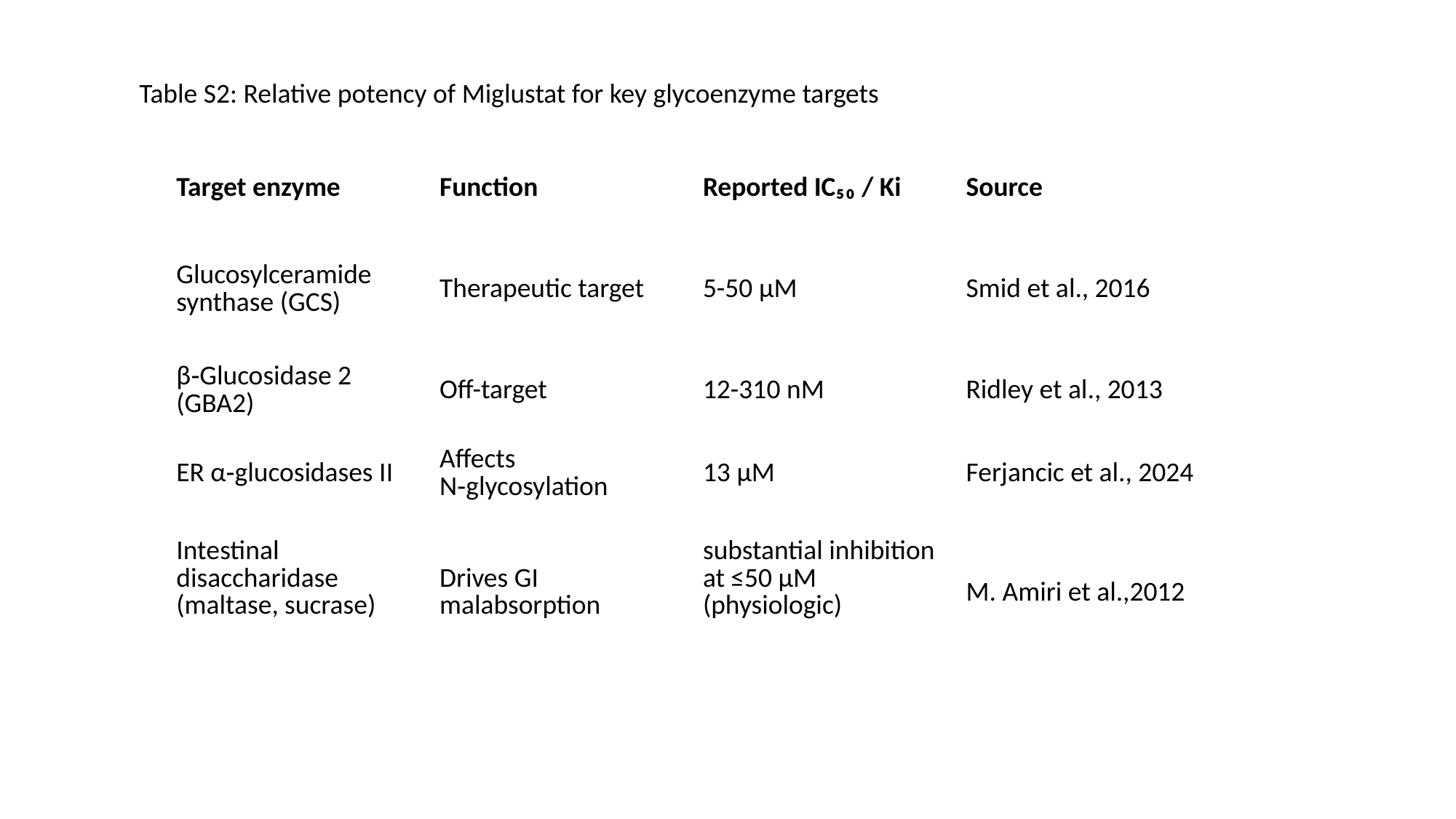

Table S2: Relative potency of Miglustat for key glycoenzyme targets
| Target enzyme | Function | Reported IC₅₀ / Ki | Source |
| --- | --- | --- | --- |
| Glucosylceramide synthase (GCS) | Therapeutic target | 5-50 μm | Smid et al., 2016 |
| β‑Glucosidase 2 (GBA2) | Off-target | 12-310 nM | Ridley et al., 2013 |
| ER α‑glucosidases II | Affects N‑glycosylation | 13 μm | Ferjancic et al., 2024 |
| Intestinal disaccharidase (maltase, sucrase) | Drives GI malabsorption | substantial inhibition at ≤50 µM (physiologic) | M. Amiri et al.,2012 |
